# Removal of a missing intrauterine contraceptive device after location through an ultrasound: a case report within a rural setting and review of literature

**DOI:** 10.1186/s40834-020-00129-2

**Published:** 2020-12-07

**Authors:** Mesele Damte Argaw, Hailemariam Segni Abawollo, Binyam Fekadu Desta, Zergu Taffesse Tsegaye, Dejene Mengistu Belete, Melkamu Getu Abebe

**Affiliations:** 1 USAID Transform: Primary Health Care, JSI Research & Training Institute, Inc., Addis Ababa, Ethiopia; 2 Mekoy Health Center, Mekoy, Antsokiya Gemza District Ethiopia

**Keywords:** Missing threads, Intra uterine contraceptive device, Ultrasonography, Ethiopia

## Abstract

**Background:**

In the last decade, (2000–2019), the modern contraceptive prevalence among married women of reproductive age (14–49 years), has increased by only 2.1%. The slow progress was due to limited access to services and myths surrounding methods held by both users and providers. This case report was identified, diagnosed and managed by a midwife working in rural health center in low resource setting. However, literature is scare on the management of missing Intra-Uterine Contraceptive Device (IUCD) thread removal services of confirmed diagnosis using Vscan or limited ultrasound services in rural health centers. The aim of reporting this case report was developed to enhance easy access to intra-uterine contraceptive method removals, which may address myths associated with difficulties of undergoing the services in rural set-up.

A 26 year-old married woman, Gravida 1 and Para 1, attended Mekoy Health Center for IUCD removal service after 7 years of protection and internally referred to limited obstetric ultrasound service room due to non- visualization of IUCD thread with Vaginal Speculum examination. An ultrasonography scan however, showed a centrally located copper-T 380A IUCD in the endometrial cavity. As a result, after dilatation of the cervix, a successful removal of the Copper-T 308A was conducted. The client received followed up care for 2 hours post-procedure and was then discharged.

**Conclusions:**

This case highlights the importance of availing diagnostic and removal services in rural set ups to mitigate myths in the community. The availability of limited obstetric ultrasound scanning services can improve the diagnoses and management of conditions in clients. The reported case shows that although, the basic infrastructure was limited, ultrasound scanning and Long Acting Reversible Contraception (LARC) trained midwives can ensure the provision of safe IUCD removal services in rural areas.

## Introduction

The global trend of modern contraceptive prevalence among married women of reproductive age has increased slowly from 55.0% (95% CI 53.7–56.3%) in 2000 to 57.1% (95% CI 54.6–59.5%) in 2019. The limited progress was explained by poor quality of available services, limited access to services, and myths against methods held by users and providers [1].

Ethiopia is the second most populous country in Africa, with an estimated population of 114 million. In the year 2017, with a total fertility rate of 4.99, the country assumed the 15th rank among twenty countries with the highest fertility rates in the world [2]. According to the Health Sector Transformation Plan (2015–2020), the country aims to achieve modern contraceptive method coverage of 80%, where, 40% of mothers will use long-acting reversible contraceptives through informed choices [3]. However, among currently married women, the modern contraceptive acceptance rate is 41.0% and only 2.0% of them use IUCDs [4]. Quality of care affected not only the uptake of IUCD services but its removal too. A misplaced IUCD usually presents as missing threads and remains asymptomatic in most cases [5]. This report is of a case of missing IUCD threads that was diagnosed and managed with the help of portable ultrasound services following a short training in a rural set up in Ethiopia.

## Case

**Fig. 1:**
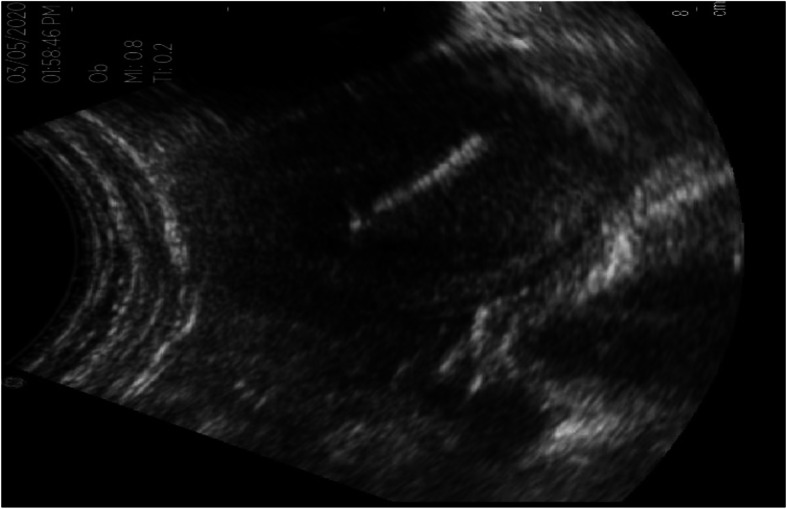
Transabdominal ultrasonic image of the intrauterine contraceptive device (copper-T 380A IUCD). Ultrasound image showed a centrally located copper-T 380A IUCD in the endometrial cavity

A 26-year-old married woman, Gravida I and Para 1, was evaluated at Mekoye Health Center’s limited obstetric ultrasound service room on May 03, 2020. The client was internally referred from the family planning service unit for non-visualization of an IUCD thread upon undergoing a vaginal speculum examination. The client attended the clinic to get an IUCD removal service and was showing no signs and symptoms of abnormal health conditions. Seven years ago, she had experienced a normal vaginal delivery and an IUCD was inserted 42 days after delivery. The client had no history of known medical or surgical health abnormalities and had no history of reported expulsion of IUCD. After providing a brief explanation of the speculum vaginal examination procedure and its purpose, the provider obtained verbal consent. The speculum examination revealed a normal looking vaginal wall and cervix. To complete the gynecological workup, a bimanual examination was performed revealing that her uterus was anteverted and was a normal non-pregnant uterus size. An ultrasonography scan however, (Fig. 1), showed a centrally located copper-T 380A IUCD in the endometrial cavity. As a result, after dilatation of the cervix, a successful removal of the IUCD was conducted. The client received followed up care for 2 hours post-procedure and was then discharged.

## Discussion

Intrauterine contraceptive device is one of the types of long-acting reversible contraceptive (LARC) methods. IUCD is a safe and effective contraceptive method and fertility of women returns promptly after removal [6]. Ethiopia - like many low-income countries - strives to address its unmet family planning need and control its population growth through promoting modern contraceptive methods [3].

According to the mini-Ethiopian Demographic and Health Survey (2019), the overall modern contraceptive prevalence rate (CPR) among married women is 41.0%, while IUCD use remains low at 2.0% [4]. The Ministry of Health and its development partners work to strategically improve quality and equity for, and access to LARC services. Some of the interventions applied to achieve this are promoting task shifting/sharing between health extension workers and facilitating community back-up services by trained clinical staff of health centers and primary hospitals [3, 4]. However, the proportion of IUCD use among LARCs is about 7.3%. One of the factors influencing uptake of IUCD services includes misconception on delays to returning to normal fertility [7]. Among the many reasons for low uptake, the delay in getting access to removal services and more specifically, patients with missing threads may create myths in the community.

The client in the reported case who was asymptomatic was attending the health center to receive an IUCD removal service. The string, which can be used to remove the IUCD, was not visible to the family planning unit’s service providers. This finding was in line with an estimation of up to 30 to 85% of patients with displaced or migrated IUCDs being asymptomatic cases [5, 8]. Mishra (2017), confer the causes of lost strings as expulsion curling and indrawing into the uterine cavity [6]. The incidence of complications of IUCD strings can be affected by several factors which includes the timing of IUCD insertion, uterine position, past history of abortion, and the operator’s experience [5]. Similarly, Mishra (2017) explored that missing strings after post-placental intrauterine contraceptive device and low segment caesarian section insertion is a common problem. They pointed out that it is important to follow up on infrequently occurring complications on a regular basis with the increasing use of IUCDs [6]. Sowmya, et al., (2016) confer the importance of performing post-insertion close follow up care after a month, subsequent 3 months and on a yearly basis for the exclusion of infection, abnormal bleeding, and the proper positioning of copper-T. In addition, IUCD users should receive proper counseling services to make them aware of the need to contact health care providers in cases where IUCD threads cannot be felt, for persistent abdominal pain and missed periods [9].

The United States Agency for International Development funded Transform: Primary Health Care Activity has enhanced access to limited obstetric ultrasound services at the primary health care, (health center level) in rural Ethiopia [10]. Cognizant of the low number of available specialized senior health professionals in the country and their maldistribution, i.e. concentration in large cities, the Activity trained midwives and other mid-level health professionals recruited from 100 health centers and supplied portable ultrasound machines [10]. This is in line with the Sikolia et al., (2017) recommendation that enhanced point of care through the provision of portable ultrasound services help healthcare providers to make evidence-based decisions for mothers [11]. A midwife working in Mekoy Health Center identified and removed the IUCD in the reported case using narrow-type forceps (Spencer Wells or alligator forceps). This procedure was in line with the Moro et al., (2015) novel ultrasound-guided procedure for successful retrieval of lost IUCDs and is well tolerated by women [12]. The use of narrow-type forceps for removal of missing threads is also referenced by Dalby et al., (2017) [13]. In addition, the availability of limited obstetric ultrasound services helps the health system to avert unnecessary referrals and reduces costs associated with referral services for clients [14].

## Conclusion

Long-acting reversible contraceptive methods are promoted to address the high unmet family planning needs among reproductive aged married women and control the population growth of low-income countries. This case highlights the importance of availing diagnostic and removal services in rural set ups to mitigate myths in the community. The availability of limited obstetric ultrasound scanning services can improve the diagnoses and management of conditions in clients. The reported case shows that although, the basic infrastructure was limited, ultrasound scanning and LARC trained midwives can ensure the provision of safe IUCD removal services in rural areas. In addition, close follow up to alleviate pain and observation of any resulting complications were carried out. After the safe removal of the IUCD, family planning counseling services were also provided.

## Supplementary information

**Additional file 1.** Consent Form for Case Reports.

## Data Availability

The datasets used and/or analysed during the current study are available from the corresponding author on reasonable request.

## Abbreviations

CPR: Contraceptive Prevalence Rate
FMOH: Federal Ministry of Health IUCD Intra-Uterine Contraceptive Device
LARC: Long-Acting Reversible Contraceptive
USAID: United States Agency for International Development
