# Supplementary material for: Removal of a missing intrauterine contraceptive device after location through an ultrasound: a case report within a rural setting and review of literature
Source: Contracept Reprod Med. 2020 Dec 7;5:23. doi: 10.1186/s40834-020-00129-2 (PMC7720470; doi:10.1186/s40834-020-00129-2)
Supplement: Supplementary file 1 — Additional file 1. Consent Form for Case Reports. [file 40834_2020_129_MOESM1_ESM.docx]

**Additional File 1**

**Consent Form for Case Reports**

**Case Report:** Removal of a missing intrauterine contraceptive device after location through an ultrasound: a case report within a rural setting and review of literature

**Principal Investigator:** Mesele Damte Argaw, Ph.D.

JSI Research & Training Institute Inc.

Contact Phone Number, E-mail, Fax

You are being asked to consider allowing Dr. Mesele Damte Argaw to use information about your diagnosis and management of missing intrauterine contraceptive device to write what is called a case report. Case reports are typically used to share new unique information experienced by one patient during his/her clinical care that may be useful for other physicians and members of a health care team. A case report may be published (in print and/or via internet dissemination) for others to read, and/or presented at a conference. This form explains the purpose of this case report. Please read this form carefully and take your time to make your decision and ask any questions that you may have.

The purpose of this case report is to inform other physicians that removal of missing IUCD in a rural health center by ultrasonography and Long Acting Reversible Family Planning Methods trained mid-level health workers helps to reduce myths by community members and health care workers about the methods.

Your information being used for this case report includes age, gravida, para, residence, history of family planning, experiences of side effect, medical and surgical health conditions.

Dr. Mesele Damte Argaw is obligated to protect your privacy and not disclose your personal information (information about you and your health that identifies you as an individual e.g. name, date of birth, medical record number). When the case report is published or presented, your identity will not be disclosed.

Although your personal information collected or obtained will be kept confidential and protected to the fullest extent of the law, there is a limited risk associated with this case report that could result in a loss of confidentiality by virtue of your unique experience.

You will not directly benefit from participating in this case report. The information that can be shared with other health care professionals, however, may improve the care that is received by others in the future.

Allowing your information to be used in this case report will not involve any additional costs to you. You will not receive any compensation.

Taking part in this case report is your choice (voluntary). You may choose not to take part or you may change your mind at any time. However, once the case report is written and published, it will not be possible for you to withdraw it. Your decision will not result in any penalty or loss of benefits to which you are entitled including the quality of care you receive.

You will be told about any new information relating to this case report that may affect you.

Your signature below means that you have read the above information about this Case Report and have had a chance to ask questions to help you understand how your information will be used and that you give permission to allow your information to be used in this case report.

If you have any questions or concerns regarding the publication/presentation of the case, or if any problems arise, please contact Mr. Binyam Fekadu at (251) 113-203501 or at 251912605658.

**SUBJECT CONSENT TO PARTICIPATE**

Case Report Title: Removal of a missing intrauterine contraceptive device after location through an ultrasound: a case report within a rural setting and review of literature

Name of Participant: W/ro Berhan Dereb

Participant/Substitute decision-maker

By signing this form, I confirm that:

- The case report has been fully explained to me and all of my questions have been answered to my satisfaction
- I have been informed of the risks and benefits, if any, of allowing my information to be used in this case report
- I have been informed that I do not have to participate in this case report
- I have read each page of this form
- I authorize access to my personal health information (medical record) as explained in this form
- I have agreed to participate in this case report

__________________________ _______________________ _____________________

Name of Participant/Substitute Signature Date

Decision-maker (print)
